# Regulation of *Bacteroides acidifaciens* by the aryl hydrocarbon receptor in IL-22-producing immune cells has sex-dependent consequential impact on colitis

**DOI:** 10.3389/fimmu.2024.1444045

**Published:** 2024-08-20

**Authors:** Chandani Mitchell, Shanieka Staley, Michal Claire Williams, Archana Saxena, Raymond Bogdon, Kasie Roark, Michele Hailey, Kathryn Miranda, William Becker, Nicholas Dopkins, Maria Marjorette Pena, Kristen M. Hogan, Maredith Baird, Kiesha Wilson, Prakash Nagarkatti, Mitzi Nagarkatti, Philip Brandon Busbee

**Affiliations:** ^1^ Department of Pathology, Microbiology, and Immunology, School of Medicine, University of South Carolina, Columbia, SC, United States; ^2^ Department of Biological Sciences, College of Arts and Sciences, University of South Carolina, Columbia, SC, United States

**Keywords:** inflammatory bowel disease, colitis, aryl hydrocarbon receptor, indole-3-carbinol, interleukin-22, innate lymphoid type 3 cells, bacteroides acidifaciens, sex differences

## Abstract

**Introduction:**

Colitis is an inflammatory bowel disease (IBD) characterized by immune cell dysregulation and alterations in the gut microbiome. In our previous report, we showed a natural product in cruciferous vegetables and ligand of the aryl hydrocarbon receptor (AhR), indole-3-carbinol (I3C), was able to reduce colitis-induced disease severity and microbial dysbiosis in an interleukin-22 (IL-22) dependent manner.

**Methods:**

In the current study, we performed single-cell RNA sequencing (scRNAseq) from colonocytes during colitis induction and supplementation with I3C and show how this treatment alters expression of genes involved in IL-22 signaling. To further define the role of IL-22 signaling in I3C-mediated protection during colitis and disease-associated microbial dysbiosis, we generated mice with AhR deficiency in RAR-related orphan receptor c (Rorc)-expressing cells (AhR^*ΔRorc*^) which depletes this receptor in immune cells involved in production of IL-22. Colitis was induced in wildtype (WT), AhR^*ΔRorc*^, and littermate (LM) mice with or without I3C treatment.

**Results:**

Results showed AhR^*ΔRorc*^ mice lost the efficacy effects of I3C treatment which correlated with a loss of ability to increase IL-22 by innate lymphoid type 3 (ILC3s), not T helper 22 (Th22) cells. 16S rRNA microbiome profiling studies showed AhR^*ΔRorc*^ mice were unable to regulate disease-associated increases in Bacteroides, which differed between males and females. Lastly, inoculation with a specific disease-associated Bacteroides species, *Bacteroides acidifaciens* (*B. acidifaciens*), was shown to exacerbate colitis in females, but not males.

**Discussion:**

Collectively, this report highlights the cell and sex-specific role of AhR in regulating microbes that can impact colitis disease.

## 1 Introduction

Inflammatory bowel disease (IBD) encompasses a spectrum of disorders within the gut that impairs the integrity of the intestinal barrier, disturbs immune and microbial homeostasis, and causes chronic or progressive inflammation in the gastrointestinal tract (GI) (1). Two well-characterized forms of IBD in human patients, Crohn’s Disease (CD) and ulcerative colitis, have shared clinical manifestations which include, but are not limited to, diarrhea, weight loss, and bloody stool with additional presentations such as anorexia, fatigue, and abdominal pain (2–4). The clinical consequences of these symptoms associated with IBD can cause dysfunction in the anus and rectum, fecal incontinence, and abnormal movement with further complications in the GI tract consisting of increased strictures, abscesses, and risk of tumor development, all having a negative impact on quality of life for diagnosed patients (5). The prevalence of IBD, which is linked to developing and urbanized countries, has continued to increase worldwide over the past decade with 25% of patients developing it before the age of 20 and the highest rates of disease in the age ranges from 20 to 60 years (6–8).

IBDs, such as colitis, have a complex etiology, but it is generally believed that in addition to genetics and environmental factors, dysregulation of the immune system and the gut microbiota play pivotal roles in disease manifestation and progression. Standard IBD treatments target the host immunity and the microbiota with the use of antibiotics, anti-inflammatory steroids, fecal material transplant (FMT) therapies, and biologics directed against inflammatory mediators such as tumor necrosis factor alpha (TNFα) (9–11). However, despite advances in IBD treatment, standard therapeutic approaches are often met with deleterious outcomes such as negative side effects after prolonged use, increased susceptibility to secondary infections associated with immune suppression, and nonresponding patients (12–14). With growing incidence, high costs associated with continuous treatment of incurable IBD patients (15), and the unmet challenges still facing standard care, identification of alternative therapies and therapeutic targets are a continued focus with respect to the IBD patient population (14, 16–18). One such therapeutic target showing promise in the IBD research field is the aryl hydrocarbon receptor (AhR), which when targeted in colitis models by various exogeneous and endogenous ligands often reduces disease severity (19–26).

AhR is a cytosolic-bound receptor and transcription factor which can facilitate the detection of metabolic, dietary, and environmental cues (27, 28). The attraction for targeting AhR in IBD-related research is due to the fact this receptor has been shown to regulate key contributing factors linked to this disease, including GI-specific immune responses and regulation of the gut microbiome (29–34). Research highlighting the regulation of interleukin-22 (IL-22) by AhR is of particular relevance to IBD as this cytokine impacts intestinal epithelial cell regeneration processes and is implicated in maintaining gut microbiome homeostasis (32, 35–39). In our previous report, we showed treatment with an AhR ligand, indole-3-carbinol (I3C), reduced disease severity and disease-associated intestinal microbial dysbiosis in murine models of colitis, which appeared dependent on IL-22 production by innate lymphoid type 3 cells (ILC3), not T helper 22 (Th22) cells (37). However, what was not established in this previous report was whether AhR was specifically driving these observations during I3C treatment and colitis, particularly as it related to increased IL-22 production by ILC3s and regulation of the gut microbiome. In addition, the previous report only determined these observations in female mice and did not address any potential sex differences, which is important given AhR is known to have sex-specific effects (40, 41).

In the current study, we continued using I3C administration to determine AhR-mediated effects on colitis and regulation of the gut microbiome with focused attention on IL-22 production. We used AhR conditional knockout mice with select deletion in Rorc-expressing cells (AhR*^ΔRorc^*) since it was previously shown these mice have deletion of AhR in T cell and ILC3 populations which are major producers of IL-22, particularly in the GI tract (42–44). Using a combination of single cell RNA sequencing (scRNAseq) and flow cytometry, we profile tissue-derived and tissue-infiltrating lymphoid cells to determine the source of AhR-dependent IL-22 production in response I3C supplementation. In doing so, we demonstrate the importance of tissue derived ILC3s in regulating colitis in both male and female mice. Furthermore, we demonstrate sex differences within microbiota composition of colitic and control mice, and Bacteroides abundances within the gastrointestinal microbiota respond to AhR signaling in a sex-specific manner. Lastly, based on our previous identification of *Bacteroides acidifaciens* (*B. acidifaciens*) as the predominant Bacteroides species increased during colitis induction in female mice (37), the effect of *B. acidifaciens* on colitis was assessed. We show that *B. acidifaciens* exacerbated disease severity in female mice induced with colitis, but not in males. In addition, inoculation of *B. acidifaciens* into germ-free (GF) female mice resulted in induction spontaneous colitis-like symptoms, revealing that this species promoted disease and was not limited to enhancing an already established disease state. The key findings from the current report are AhR can regulate IL-22 signaling in specific cell types during colitis to alter the gut microbial profile which have consequential and sex-dependent impacts on disease severity.

## 2 Materials and methods

### 2.1 Animals

Adult specific pathogen free (SPF) female and male mice (8-12 weeks old) were purchased from The Jackson Laboratory (Bar Harbor, ME). Germ-free (GF) female mice (8-12 weeks old) used in studies were purchased from Charles River Laboratories (Raleigh, NC). This study used WT Balb/c (Strain #:000651), WT C57BL/6 (SPF: Strain #000664, GF: Strain #574), AhR*^ΔRorc^*, and littermate (LM) controls. AhR^ΔRorc^ and LM mice were on a C57BL/6 background. AhR*^ΔRorc^* mice were generated by breeding Ahrtm3.1Bra/J (strain#: 006203) and B6.FVB-Tg(Rorc-cre)1Litt/J (Strain #:022791) to deplete AhR specifically in Rorc-expressing cells. Controls for the conditional AhR mice were either LM or WT C57BL/6 mice which were age and sex matched. All breeding was conducted in-house at the animal facilities at the University of South Carolina School of Medicine (USC SOM). Confirmation of the conditional knockout included genotyping by PCR analysis of their DNA collected from tail snips. DNA was isolated from samples using the DNeasy Blood & Tissue Kit from Qiagen (Hilden, Germany). Primers designed by the Jackson Laboratory and purchased from IDT Technologies (Coralville, IA) were used for genotyping (**Table 1**). All mice were housed and cared for at the USC SOM Animal Facility under SPF conditions, with the exception of GF mice which were housed under strict GF conditions supervised by the Mouse Experimentation and Gnotobiotic Core Facility (MEGCF) at USC. The parameters for their care consisted of a standard temperature regulated room (23°C) with 45% humidified conditions, a 12:12-h light-dark cycle, and all mice were provided standard rodent chow and water ad libitum, unless otherwise noted. All experiments and procedures involving research mice were approved by the USC SOM Institutional Animal Care and Use Committee (IACUC) under the following protocol numbers: 2467101451090319, 2557101628062421.

**Table 1:** Primers used for PCR.

Primer	Forward	Reverse
AhR	CAGTGGGAATAAGGCAA GAGTGA	GGTACAAGTGCACATG CCTGC
Rorc	TTCCGGTTATTCAACTT GCAC	TGTCCTGGGCTACCCT ACTG
RorcIP	CTAGGCCACAGAATTGA AAGATCT	GTAGGTGGAAATTCTA GCATCATCC
GAPDH	AACAGCAACTCCCACT CTTC	CCTGTTGCTGTAGCCGT ATT
B. acidifaciens	GTATGGGATGGGGATGC GTT	CTGCCTCCCGTAGAGTT TGG
Eubacteria	ACTCCTACGGGAGGCAG CAGT	ATTACCGCGGCTGCTG GC

### 2.2 Induction of colitis models and I3C administration

2,4,6-trinitrobenzene sulfonic acid (TNBS, Sigma Aldrich, St. Louis, MO) and dextran sodium sulfate (DSS, MP Biomedicals, Santa Ana, CA) colitis models were induced in mice as previously described (37). For the TNBS model, 100 microliters containing 1mg TNBS (5% w/v, Sigma-Aldrich, St. Louis, MO) in 50% ethanol vehicle was intrarectally (i.r.) injected into Balb/cJ mice under anesthetized conditions with 5% isoflurane. DSS colitis was induced in C57BL/6 mice by giving 3% DSS (molecular weight: 36,000–50,000) in their drinking water ab libitum for 7 days, followed by regular drinking water for the remainder of the experiment (10-14 days). For treatment groups, I3C (40 mg/kg; Sigma-Aldrich, St. Louis, MO) was dissolved in an appropriate vehicle of 0.05% dimethyl sulfoxide (DMSO, Sigma-Aldrich, St. Louis, MO) and corn oil (Sigma-Aldrich, St. Louis, MO) and administered through the intraperitoneal (i.p.) route within 1 hour after colitis induction. For the TNBS model, I3C was administered every day after colitis induction until experimental endpoint (4-6 days). For the DSS model, I3C was injected every other day after colitis induction until experimental endpoint (10-14 days). For control groups, injections of appropriate vehicles were used.

### 2.3 Assessment of colitis disease severity

The weight of mice was recorded daily and expressed as percent weight loss or gain over experimental timeline from the starting body weight. Colonoscopies were conducted 24 hours prior to experimental endpoints using a Tele Pack Vet X LED endoscope (Karl Storz, El Segundo, CA). Colonoscopy scores were based on parameters as described previously, which included scoring criteria based on the following factors: perianal findings, wall transparency, intestinal bleeding, and focal lesions (45). At experimental endpoints, mice were euthanized by inhalation of a lethal overdose of isoflurane in accordance with approved protocols. After euthanasia, colon lengths were measured starting from the bottom of the cecum superior to the proximal colon ending at the rectum. In addition, blood was collected from the mice (~100 µl) using the retroorbital method for analysis of blood as previously described (37). Blood diagnostics was performed on 10 µl of whole blood using a Vetscan HM5 Hematology analyzer (Zoetis, Parsippany, NJ). Data outputs included a full blood diagnostic panel and results presented herein included concentrations or percentages of white blood cells (WBCs), lymphocytes (LYM), monocytes (MON), and neutrophils (NEU).

### 2.4 Cell isolation from the colon tissue

Cells were isolated from colons of experimental mice as previously described (46). In brief, whole colons were excised from experimental mice after euthanasia. Luminal contents and mucus were removed by combination of mechanical extraction and flushing with 1x PBS (VWR, Radnor, PA). Cleansed colon tissues were cut into 0.5 cm pieces and incubated in sterile 1x HBSS (without Ca^2+^ and Mg^2+^) containing 3% FBS, 10 mM EDTA, and 5 mM DL-dithiothreitol or DTT (VWR, Radnor, PA). Samples were shaken for 30 minutes at 37°C prior filtering using sterile 100 µM filters (VWR, Radnor, PA). Samples were placed on ice for 10 minutes to allow sedimentation, and the upper supernatant fraction was isolated as the intra-epithelial cell portion. Remaining tissue was incubated and shaken (45 minutes at 37°C) at least 3 times in 1x HBSS (with Ca^2+^ and Mg^2+^, VWR, Radnor, PA) containing 3% FBS (VWR, Radnor, PA), 1% L-glutamine (Sigma Aldrich, St. Louis, MO), 1% penicillin/streptomycin (VWR, Radnor, PA), 10 mM HEPES (VWR, Radnor, PA), 0.5 mg/ml collagenase D (Roche, Indianapolis, IA), 0.5 mg/mL Dispase (Sigma Aldrich, St. Louis, MO), 0.04 mg/mL DNase I (Sigma Aldrich, St. Louis, MO). Samples were filtered using 70 µM filters into cold 1x PBS (VWR, Radnor, PA) prior to layering on a 40%/80% Percoll (VWR, Radnor, PA) gradient to spin for 620 x g for 20 minutes with low acceleration and no brake. Lamina propria-derived cells were collected at the interface of the Percoll gradient and washed twice in 1x PBS containing 3% FBS prior to further downstream processing.

### 2.5 Flow cytometry analysis

Processing of isolated lamina propria-derived colonocytes for flow cytometry analysis was performed as previously described (46). Cells were washed in cold 1x PBS (VWR, Radnor, PA) and stained with Zombie Aqua Fixable Viability kit from Biolegend (San Jose, CA) following instructions from the manufacturer. Cells were then washed once before incubating and blocking with TruStain FcX anti-mouse CD16/32 (Biolegend, San Jose, CA). After blocking, cells were fixed and permeabilized with BD Transcription Factor Buffer (BD Biosciences, San Jose, CA). Fluorescently tagged antibodies used for cellular profiling are summarized in **Table 2**. Flow cytometry was performed using a BD FACSCelesta (BD Biosciences, San Jose, CA) and downstream analysis was completed using FlowJo software (BD Biosciences, San Jose, CA).

**Table 2:** Antibodies used for flow cytometry.

Antibody	Clone	Source
Alexa Fluro 700 anti-mouse lineage cocktail	17A2/RB6-8C5/RA3-6B2/Ter-119/M1/70	Biolegend
Brillant Violet 785 anti-mouse CD4	GK1.5	Biolegend
APC/Cy7 anti-mouse CD45	30-F11	Biolegend
FITC anti-mouse CD90.2 (Thy-1.2)	53-2.1	Biolegend
Alexa Fluro 647 anti-mouse IL-22	Poly5164	Biolegend
BD Horizon BV650 anti-mouse Rorγt	Q31-378	BD Biosciences

### 2.6 Single cell RNA sequencing

For scRNAseq, intra-epithelial and lamina propria colonocyte fractions using isolation methods detailed above were combined at a 1:1 ratio as previously described (46). TC20 Automated Cell Counter (BioRad, Hercules, CA) was used to count cells and measure viability. Samples used for scRNAseq had at least 80% viability before being processed further. Processed cells were loaded to target 1,000 cells per lane into the Chromium Controller (10x Genomics, Pleasanton, CA). Libraries were processed using the Chromium v2 single-cell 3′ RNA-seq reagent kit (10x Genomics, Pleasanton, CA). Sequencing of the libraries was completed using NextSeq 550, manufactured by Illumina (San Diego, CA), and the depth of the sequences for each cell was read at 40k-60k. Following sequencing, FASTQ files were produced using the Cell Ranger version 2 pipeline (10x Genomics, Pleasanton, CA) with reads aligned to the mm10 genome and a summary of read counts per gene for each single cell was provided. Data aggregation and analysis processing was done using R scripts packaged with Seurat suite (version 3.0) (47) and Loupe Browser (10x Genomics, Pleasanton, CA). Identification of cell clusters was determined by evaluating top gene expression (log2) and inputting into PanglaoDB (48).

### 2.7 Microbial profiling with 16S rRNA and PCR

Genomic DNA was isolated from the colonic flushes collected from experimental mice using the QIAamp Fast DNA Stool kit from Qiagen (Germantown, MD). Determination of the DNA concentration was conducted using a NanoDrop 2000c spectrophotometer (Thermo Fisher Scientific, Waltham, MA). 16S rRNA sequencing of the V3/V4 region using Illumina MiSeq platform (San Diego, CA) was performed as previously described (37). Raw sequencing data generated from Illumina-generated fastq files was then analyzed using Nephele, an online software provided by the National Institutes of Health (NIH) (49). Mapping files were uploaded into both DADA2 and QIIME2 pipelines where the resulting outputs were obtained and graphed, to include operational taxonomic units (OTUs), alpha diversity using Shannon, and beta diversity as principal components (PCs). Samples were excluded if they failed to reach at least 10,000 reads during sequencing. Nephele-generated data output was visualized using GraphPad Prism software version 10.1.2 (Boston, MA, USA). For PCR validation studies, DNA was prepped for PCR using a QuantiFast SYBR Green PCR Master Mix (Qiagen, Germantown, MD) based on the manufacturer’s instructions. Samples were run on a BioRad CFX96 qPCR system (Hercules, CA). Primers used for microbial PCR analysis are detailed in **Table 1**. Fold change calculations for bacteria, which were normalized to Eubacteria 16S rRNA primers, was determined using the ΔΔCT method as previously described (37).

### 2.8 *B. acidifaciens* studies

*B. acidifaciens* (DSM 15896, Leibniz Institute, Science Campus Braunschweig-Süd, Germany) was cultured in either Anaerobe Systems Chopped Meat Medium with Carbohydrates (CMC, Cat. No. NC0335925, Fisher Scientific, Waltham, MA) or on sterile Blood Agar plates with 5% sheep’s blood and penicillin (VWR, Radnor, PA) at 37°C. Anaerobic conditions were maintained using a BD GasPak chamber with GasPak EZ Anaerobe Gas Generating Pouch System with indicator (BD Biosciences, San Jose, CA). Concentration was determined by counting the number of colony-forming units (CFUs) after blood agar plating or by using optical density (OD) readings obtained by spectrophotometer. To study the effect of *B. acidifaciens* on DSS-induced SPF colitis mice or GF mice, mice were inoculated with bacteria via oral gavage in 100 µl 1x PBS (VWR, Radnor, PA) at a concentration of 1 x 10^9^ CFUs, as previously described (50). In both SPF and GF studies, inoculation occurred on two days (Day 0 and Day 7).

### 2.9 Statistical analysis

GraphPad Prism software version 10.1.2 (Boston, MA, USA) was used for all statistical analysis unless otherwise indicated. When comparing 3 or more groups with one variable, one-way analysis of variance (ANOVA) and Tukey’s *post hoc* multiple comparisons tests were used. When comparing 3 or more groups with two variables (example: percent weight loss/gain over multiple days), two-way ANOVA and Dunnett’s *post hoc* multiple comparisons tests were used. For comparisons between only 2 groups, an unpaired, two-tailed standard student’s t-test was used. Statistical significance was determined by having a p-value (p) at least less than 0.05.

## 3 Results

### 3.1 I3C administration impacts cell-specific expression of IL-22 and its receptor components during TNBS-induced colitis

**Figure 1:**
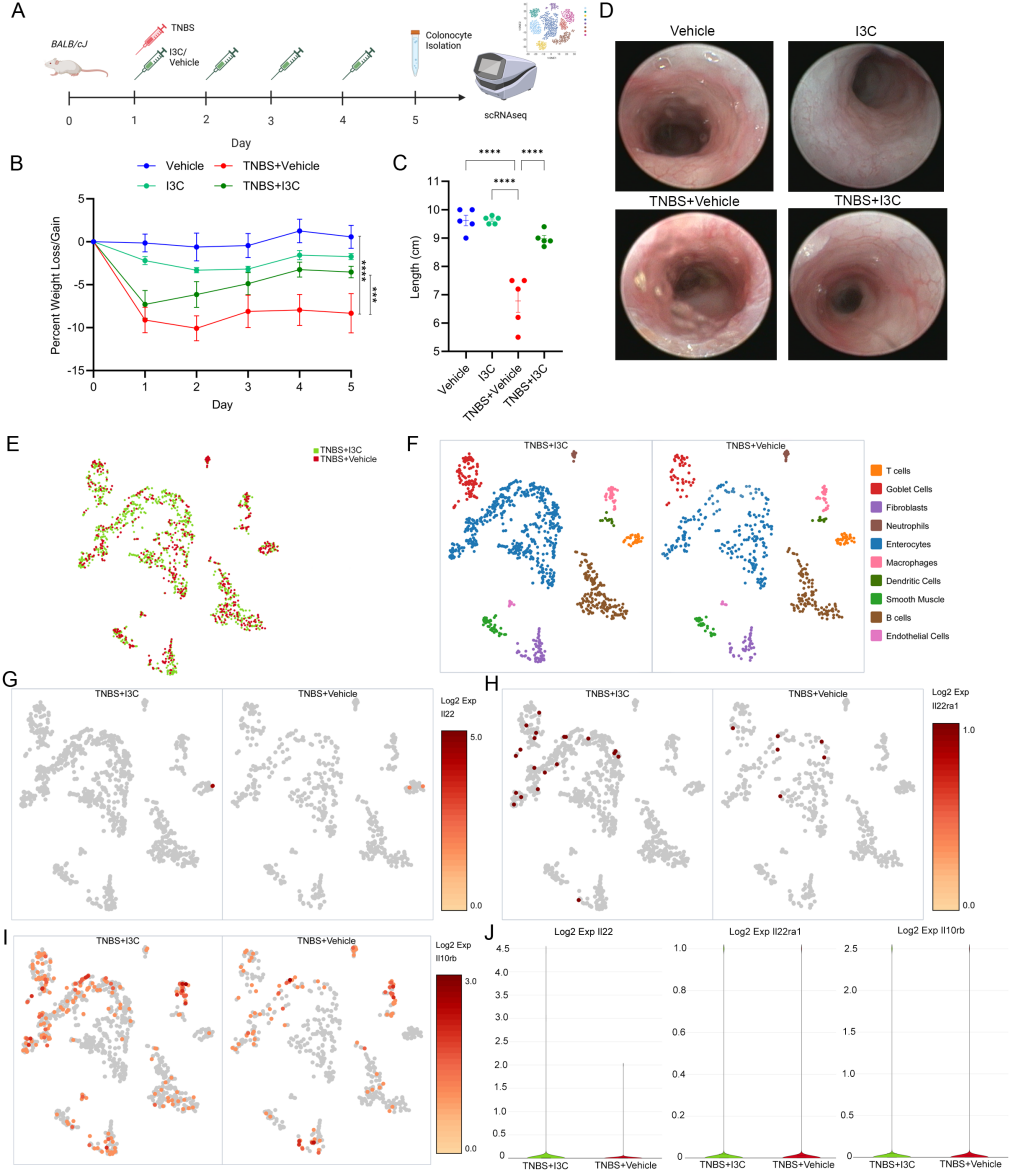
Treatment with I3C impacts cell-specific alterations in IL-22 and IL-22 receptor expression. **(A)** TNBS colitis was induced in female Balb/cJ mice (8-10 weeks) as described in detail in Material and Methods, and the efficacy of treatment with I3C was tested prior to colonocyte isolation and scRNAseq in the following groups: Vehicle (n=5), I3C (n=5), TNBS+Vehicle (n=5), TNBS+I3C (n=5). Illustration created with Biorender. Disease parameters assessed included percent weight loss **(B)** colon length **(C)** and representative colonoscopies **(D)**. **(E)** UMAP representing scRNAseq run on 1000 colonocytes from disease (TNBS+Vehicle, red) and treated (TNBS+I3C, green) groups. Data was aggregated for comparison purposes using Seurat packages and visualized by Loupe Browser (10x Genomics). **(F)** Cell clustering identification within experimental groups was determined by gene expression profiles and visualized using Loupe Browser. Log2 expression was determined between experimental groups for IL-22 **(G)**, and IL-22 receptor components: IL-22ra1 **(H)** and IL-10rb **(I)**. **(J)** Violin plots comparing overall cellular Log2 expression between disease (TNBS+Vehicle, red) and treated (TNBS+I3C, green) samples for IL-22 (left), IL-22ra (center), and IL-10rb (right). Error bars equal the standard error mean (SEM). For dot plots, significance was determined using one-way ANOVA and Tukey’s multiple comparisons test. For weight data over time, two-way ANOVA and Dunnett’s multiple comparisons test was used to determine significance (***p<0.005, ****p<0.001).

A replication study showing the efficacy of I3C treatment in the TNBS colitis mice model was conducted in female Balb/c mice as previously described (24, 37) and illustrated in **Figure 1A** prior to prepping colonocyte isolation for scRNAseq. As shown, treatment of colitis mice with I3C (TNBS+I3C) significantly reversed many of the clinical parameters present in disease controls (TNBS+Vehicle), which included weight loss (**Figure 1B**) and colon shortening (**Figure 1C**). In addition, I3C treatment reduced overall damage to the colon as result of colitis induction which was assessed by colonoscopy to include disease clinical manifestations such as colon wall thinning, ulceration development, and tissue sloughing (**Figure 1D**). A small scale scRNAseq study targeting 1000 cells was conducted on a combined intra-epithelial and lamina propria-derived colonocyte fraction from select experimental groups, mainly disease controls (TNBS+Vehicle) and treatment (TNBS+I3C) (**Figure 1E**). Ten different cell cluster populations were identified, which included the following: T cells, goblet cells, fibroblasts, neutrophils, enterocytes, macrophages, dendritic cells, smooth muscle, B cells, and endothelial cells (**Figure 1F**). After cell cluster identification, expression of molecules involved in IL-22 signaling was evaluated to include the IL-22 cytokine (**Figure 1G**) and its heterodimer receptor comprised of IL-22ra1 (**Figure 1H**) and IL-10rb (**Figure 1I**) (51). When evaluating overall cellular expression, only IL-22 appeared to have a substantial increase after I3C treatment, while IL-22ra1 and IL-10rb appeared to be relatively the same (**Figure 1J**). When focusing on expression in identified cellular subsets, IL-22 was essentially only present in the T cell cluster, with a two-fold increase in expression when comparing treated to the disease control (**Supplementary Figure 1**). IL-22ra1 expression was detected mainly in goblet cells and enterocytes for both treated and disease controls at similar levels, but interestingly it was noted that I3C treatment increased expression of this receptor component in fibroblasts, which was not present in colitis controls (**Supplementary Figure 2**). IL-10rb was drastically decreased in fibroblasts and dendritic cells but increased in goblet cells and endothelial cells after treatment with I3C when compared to disease controls (**Supplementary Figure 3**). Collectively, these data showed that I3C treatment during colitis was able to alter expression of IL-22 and its receptor in a cell-specific manner, with the most notable change being overall IL-22 production regulated to the cluster most prominently identified as T cells or T cell-like.

### 3.2 Select deletion of AhR in Rorc-expressing cells prevents I3C-mediated reduction in DSS-induced colitis severity

**Figure 2:**
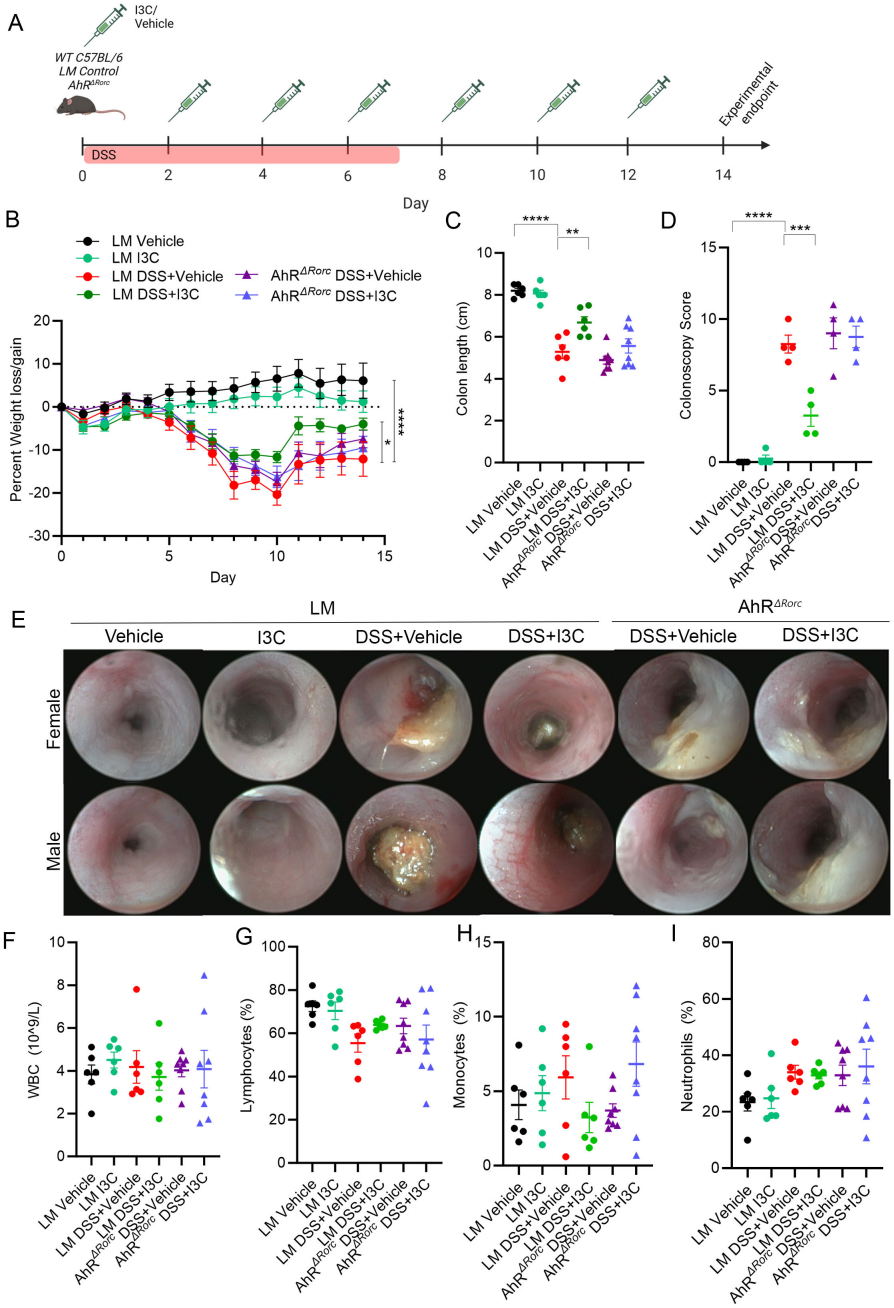
Deletion of AhR in Rorc-expressing cells lessens I3C effectiveness in minimizing DSS-induced colitis severity. **(A)** DSS colitis was induced in male and female C57BL/6 mice (8-10 weeks) as described in detail in Material and Methods, and the efficacy of treatment with I3C was tested AhR^ΔRorc^ and LM control mice divided in the following groups: LM Vehicle (n=6), LM I3C (n=6), LM DSS+Vehicle (n=6), LM DSS+I3C (n=6), AhR^ΔRorc^ DSS+Vehicle (n=8), and AhR^ΔRorc^ DSS+I3C (n=8). Experimental groups included equal number of female and male mice. Illustration created with Biorender. Disease parameters assessed included percent weight loss **(B)** colon length **(C)**, along with colonoscopy scores (n=4 mice per group; equal number female and male) **(D)** and representative colonoscopy images **(E)**. Blood panel analysis was assessed with Vetscan for WBCs **(F)**, lymphocytes **(G)**, monocytes **(H)**, and neutrophils **(I)**. For dot plots, significance was determined using one-way ANOVA and Tukey’s multiple comparisons test. For weight data over time, two-way ANOVA and Dunnett’s multiple comparisons test was used to determine significance (** p<0.01, ***p<0.005, ****p<0.001).

As IL-22 signaling components were confirmed to be impacted by I3C treatment, mice with select deletion of AhR in Rorc-expressing cells (AhR*^ΔRorc^*) were developed using the cre-flox method, as this gene is prevalent in IL-22 producing immune cells such as T cells and ILC3s (42). Since AhR*^ΔRorc^* mice and their parent strains were on a C57BL/6 background, which are more resistant to acute TNBS-induced colitis (52–54), induction of colitis was administered using the DSS method and treatment regimen with I3C was performed as previously described (24, 37) and illustrated in **Figure 2A**. Experimental groups consisted of two sex-mixed cohorts, compromised of either AhR*^ΔRorc^* or their LM counterpart controls. LM controls included LM+Vehicle, LM+I3C, LM+DSS+Vehicle, and LM+DSS+I3C. AhR*^ΔRorc^* groups consisted of either AhR*^ΔRorc^* +DSS+Vehicle and AhR*^ΔRorc^* +DSS+I3C. Results showed that while LM colitis mice treated with I3C (LM+DSS+I3C) lost significantly less weight compared to their disease controls (LM+DSS+Vehicle), AhR*^ΔRorc^* colitis mice treated with I3C (AhR*^ΔRorc^* +DSS+I3C) had no significant difference in weight loss when compared to their respective disease controls (**Figure 2B**). Additionally, unlike LM colitis mice treated with I3C, AhR*^ΔRorc^* colitis mice administered treatment exhibited shorter colon lengths (**Figure 2C**), higher colonoscopy scores (**Figure 2D**), and substantial tissue damage and scarring was evident in colonoscopy images, comparable to their disease controls (**Figure 2E**). To determine if this inflammation was present in the systemic circulation, cell profiling of whole blood was evaluated focusing on important immune cell populations, such as white blood cells (WBCs), lymphocytes, monocytes, and neutrophils (**Figures 2F–I**). The results, which were noted to be highly variable, indicated a lack of statistically significant differences between all groups, suggesting more localized responses were being affected. In summation, AhR*^ΔRorc^* colitis mice treated with I3C mirrored LM and AhR*^ΔRorc^* disease controls in both sexes, if not with slightly more severe colitis phenotype, highlighting the importance of AhR in these cell types when responding I3C administration.

### 3.3 Loss of AhR in Rorc-expressing cells leads to reduction of IL-22 production in ILC3s, not Th22 cells, during DSS-induced colitis

**Figure 3:**
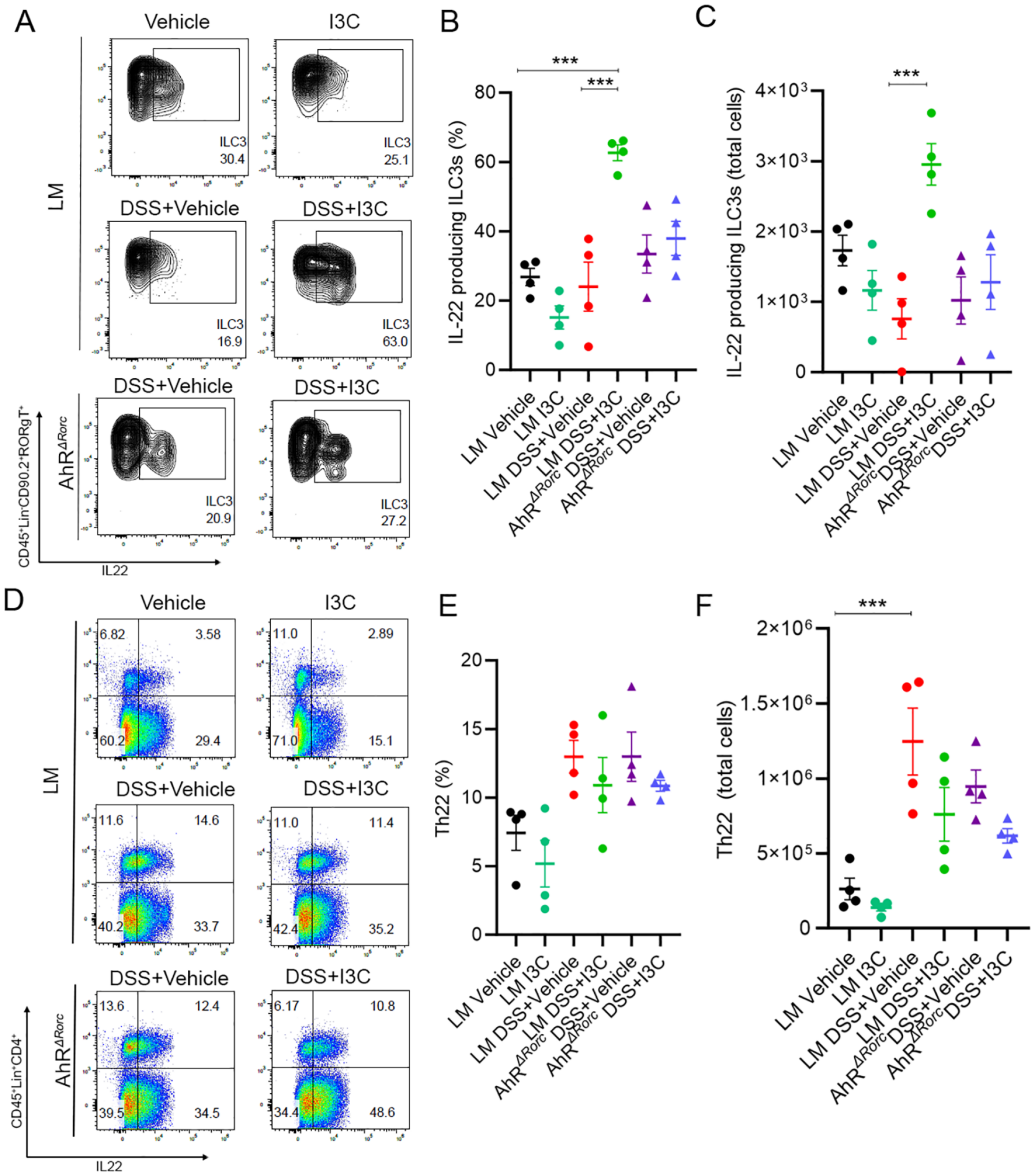
Loss of AhR in Rorc-expressing cells leads to decrease IL-22 production by ILC3s, not Th22 cells. Flow cytometry was performed on colonocytes isolated from the lamina propria from the following groups: LM Vehicle (n=4), LM I3C (n=4), LM DSS+Vehicle (n=4), LM DSS+I3C (n=4), AhR^ΔRorc^ DSS+Vehicle (n=4), and AhR^ΔRorc^ DSS+I3C (n=4). Groups consisted of equal numbers of female and male samples. **(A)** Representative flow plots for ILC3s, defined as CD45^+^Lin^-^CD90.2^+^RORgT(Rorγt)^+^IL-22^+^. **(B)** Dot plot depicting percentage of IL-22 producing ILC3s. **(C)** Dot plot depicting total cell number of IL-22 producing ILC3s. **(D)** Representative flow plots for Th22 cells, defined as CD45^+^Lin^+^CD4^+^IL-22^+^. **(E)** Dot plot depicting percentage of Th22 cells. **(F)** Dot plot depicting total cell number of Th22 cells. Error bars equal the standard error mean (SEM). For dot plots, significance was determined using one-way ANOVA and Tukey’s multiple comparisons test. (***p<0.005).

Previously, we published results showing I3C treatment during colitis led to increased production of IL-22 specifically in ILC3s, not Th22, and this was important since neutralization of IL-22 negated I3C-mediated protective effects (37). Inasmuch, ILC3 and Th22 phenotyping by flow cytometry in LM and AhR*^ΔRorc^* mice to assess IL-22 production was performed using a similar gating strategy (**Supplementary Figure 4**). Results showed while there was a significant increase in IL-22 producing ILC3s for LM disease mice given I3C treatment (LM+Colitis+I3C) compared to their respective disease controls (LM+Colitis+Vehicle), AhR*^ΔRorc^* mice failed to respond in a similar fashion (**Figures 3A–C**). In contrast, while DSS colitis led to increased IL-22 production by Th22 cells, there were no significant differences between LM and AhR*^ΔRorc^* colitis mice treated with or without I3C (**Figures 3D–F**). Based on these results, two important observations can be made. First, it appears AhR is important for the production of IL-22 in ILC3s after I3C treatment, regardless of sex. In addition, while IL-22 production by Th22 is affected by colitis induction, this process appears to be independent of AhR, at least in response to an AhR ligand like I3C.

### 3.4 Loss of AhR in Rorc-expressing cells prevents sex-specific regulation of Bacteroides during DSS-induced colitis

**Figure 4:**
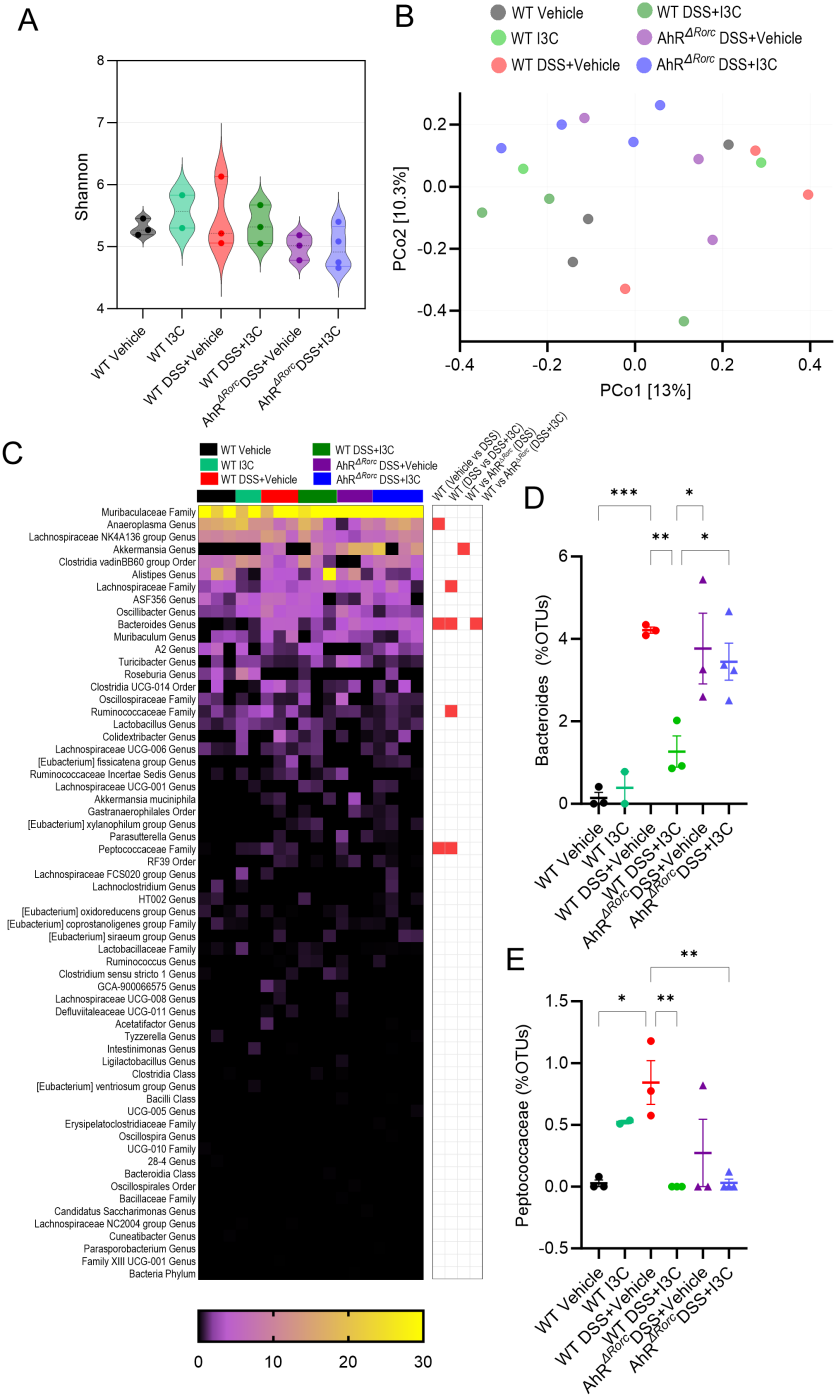
Loss of AhR in Rorc-expressing cells prevents regulation of Bacteroides during colitis after I3C treatment in female mice. 16S rRNA was performed on colonocytes isolated from the lamina propria of female mice from the following experimental groups: LM Vehicle (n=3), LM I3C (n=2), LM DSS+Vehicle (n=3), LM DSS+I3C (n=3), AhR^ΔRorc^ DSS+Vehicle (n=4), and AhR^ΔRorc^ DSS+I3C (n=4). One sample in the AhR^ΔRorc^ DSS+Vehicle group was excluded since it fell below the threshold of 10,000 reads. **(A)** Shannon index for alpha diversity. **(B)** Principal Component Analysis (PCoA) for beta diversity. **(C)** Heatmap depicting percent OTUs of most significantly altered bacterial genera (left-side); Heatmap depicting significantly altered bacteria genera when comparing two different groups (right-side; significance determined with unpaired, two-tailed t test; box highlighted in red denotes significance as p<0.05). Dot plots are shown depicting percent OTUs for **(D)** Bacteroides and **(E)** Peptococcaceae. Error bars equal the standard error mean (SEM). For dot/violin plots, significance was determined using one-way ANOVA and Tukey’s multiple comparisons test unless otherwise indicated. (*p<0.05, **p<0.01, ***p<0.005).

To investigate whether the AhR-ILC3-IL22 pathway was necessary in I3C-mediated prevention of microbial dysbiosis caused by colitis, 16S rRNA bacterial profiling was performed on fecal samples from female WT C56BL/6 and AhR*^ΔRorc^* experimental mice induced with or without colitis and/or treated with I3C. As detailed in our previous publication (24), WT mice were considered a more appropriate control for this initial screening experiment since LM mice have the low affinity AhR (*Ahr^d^*) present in all cells due to the AhR locus insert originating from 129SvJ mice to create the Ahrtm^3.1Bra^/J parent strain, while C57BL/6 normally contain the high affinity AhR receptor (*Ahr^b^*). Shannon diversity index revealed there was no significant differences in the alpha diversity among the experimental groups, though it was noted that colitis groups, and particularly AhR*^ΔRorc^* mice, had trends towards decreased diversity (**Figure 4A**). Beta diversity showed that experimental groups tended to cluster within their respective groups, though there was notable variability among samples even within their own groups (**Figure 4B**). The OTU heatmap depicts the top bacterial genera identified by sequencing amongst the different experimental groups (**Figure 4C**). Among these bacteria genera, only three were found to be significantly different when comparing WT disease and vehicle control, which included Anaeroplasma (Genus), Bacteroides (Genus), and Peptococcaceae (Family). Among those, only Bacteroides (**Figure 4D**) and Peptococcaceae (**Figure 4E**) were also significantly different between WT disease mice and those treated with I3C. Interestingly, only Bacteroides was found to be significantly different when comparing WT and AhR*^ΔRorc^* groups. Specifically, Bacteroides was increased in WT colitis mice and reduced after I3C treatment, but AhR*^ΔRorc^* colitis mice continued to have high Bacteroides even after I3C treatment. This strongly suggests regulation of colitis-associated Bacteroides in female mice was dependent on AhR expression in Rorc-expressing.

**Figure 5:**
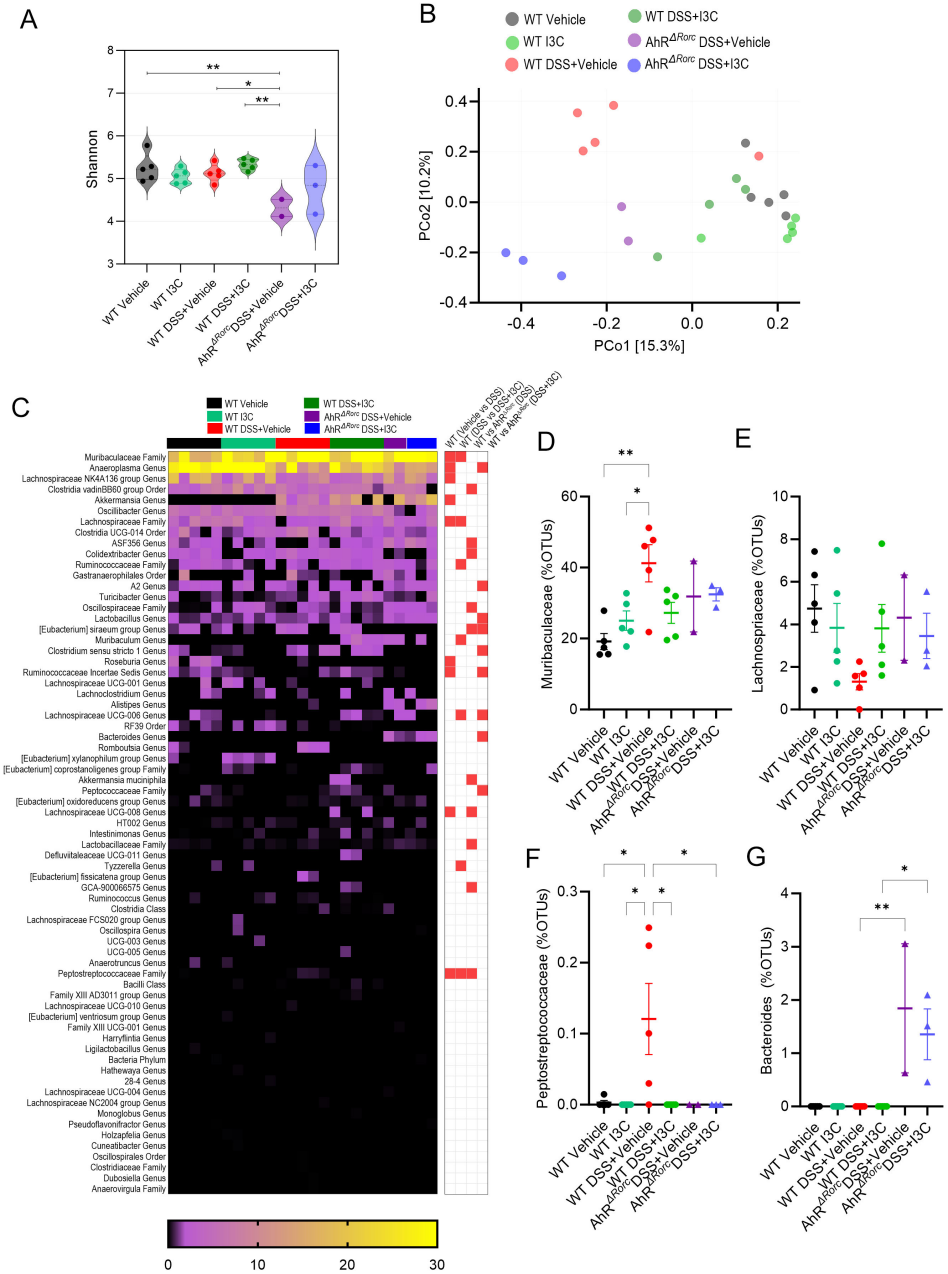
Loss of AhR in Rorc-expressing cells leads to increase in Bacteroides during colitis in male mice, which is not affected by I3C treatment. 16S rRNA was performed on colonocytes isolated from the lamina propria of male mice from the following experimental groups: LM Vehicle (n=5), LM I3C (n=5), LM DSS+Vehicle (n=5), LM DSS+I3C (n=5), AhR^ΔRorc^ DSS+Vehicle (n=3), and AhR^ΔRorc^ DSS+I3C (n=3). One sample in the AhR^ΔRorc^ DSS+Vehicle group was excluded since it fell below the threshold of 10,000 reads. **(A)** Shannon index for alpha diversity. **(B)** Principal Component Analysis (PCoA) for beta diversity. **(C)** Heatmap depicting percent OTUs of most significantly altered bacterial genera (left-side); Heatmap depicting significantly altered bacteria genera when comparing two different groups (right-side; significance determined with unpaired, two-tailed t test; box highlighted in red denotes significance as p<0.05). Dot plots are shown depicting percent OTUs for **(D)** Muribaculaceae, **(E)** Lachnospriaceae, **(F)** Peptostreptococcaceae, and **(G)** Bacteroides. Error bars equal the standard error mean (SEM). For dot/violin plots, significance was determined using one-way ANOVA and Tukey’s multiple comparisons test unless otherwise indicated. (*p<0.05, **p<0.01).

To investigate any potential sex differences, gut microbial profiling was repeated in male mice with the similar experimental groups (**Figure 5**). Results showed alpha diversity in the AhR*^ΔRorc^* colitis male mice was significantly less than in the WT controls or those treated with I3C (**Figure 5A**). Unlike with the female samples examined, beta diversity demonstrated distinct clustering of samples within their respective groups, with WT colitis mice treated with I3C clustering similarly with controls, which was not the case with AhR*^ΔRorc^* treated colitis mice, which clustered more closely with their disease control counterparts (**Figure 5B**). Additionally, several significantly different comparisons were observed in the top bacterial genera identified by OTU percent abundances (**Figure 5C**). Of these significantly altered comparisons between experimental groups, the most striking were the Muribaculaceae Family (**Figure 5D**), the Lachnospriaceae Family (**Figure 5E**), the Peptostreptococcaceae Family (**Figure 5F**), and the Bacteroides Genus (**Figure 5G**). Despite these differences, Muribaculaceae, Lachnospriaceae, and Peptostreptococcaceae did not have clear differences when comparing WT colitis treated or untreated mice with AhR*^ΔRorc^* counterparts, suggesting AhR did not control regulation of these bacteria genera, or at least not in the context of I3C response to enhanced IL-22 by ILC3s. Most interesting of all the findings was unlike females, male WT mice did not have a significant amount of Bacteroides, whether in the disease or treated states. However, Bacteroides was significantly increased only in AhR*^ΔRorc^* mice. Considering the female data, the combined results suggest AhR expression in Rorc-expressing cells, and by extension the ability to stimulate IL-22 production in ILC3s after I3C treatment, was crucial in regulating Bacteroides, which seemed to be more prominent in females when compared to males in the context of colitis.

### 3.5 B. acidifaciens can exacerbate DSS-induced colitis severity and induce colitis-like phenotype in female mice, but not male

**Figure 6:**
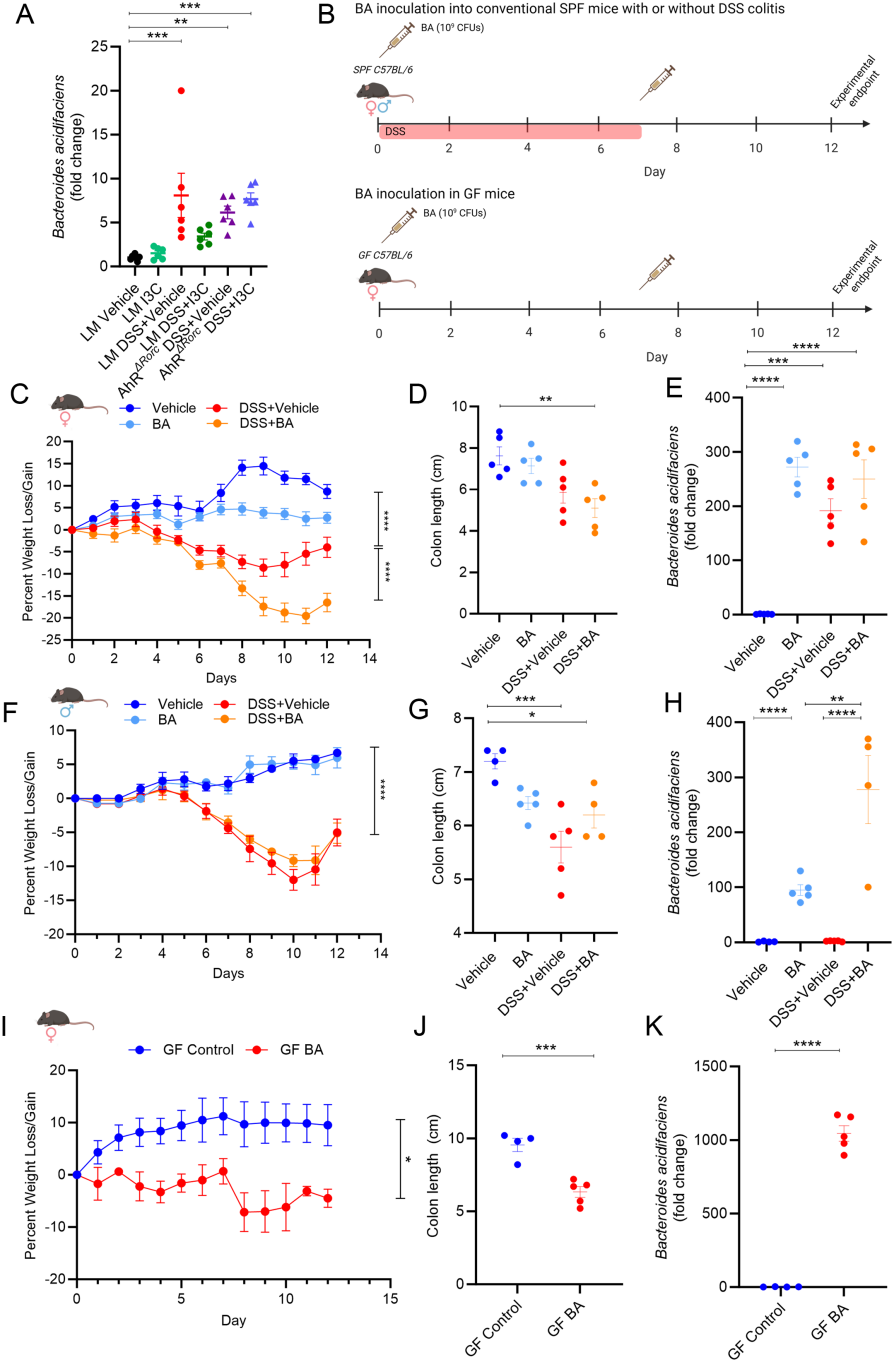
*B. acidifaciens* affects colitis in female, but not male mice. **(A)** Dot plot depicting PCR results evaluating *B. acidifaciens* from colonic content collected from female mice in the following experimental groups: LM Vehicle (n=6), LM I3C (n=6), LM DSS+Vehicle (n=6), LM DSS+I3C (n=6), AhR^ΔRorc^ DSS+Vehicle (n=6), and AhR^ΔRorc^ DSS+I3C (n=6). **(B)** Illustration depicting experimental design inoculating *B. acidifaciens* (BA) into SPF (top) and GF (bottom) experimental cohorts. Created with Biorender. **(C–E)** Results from BA inoculation studies in SPF female mice with DSS-induced colitis in the following groups: Vehicle (n=5), BA (n=5), DSS+Vehicle (n=5), DSS+BA (n=5). **(C)** Graph depicting weight loss/gain over time. **(D)** Dot plot depicting colon length. **(E)** Dot plot depicting fold change of *B. acidifaciens* compared to Vehicle control as assessed by PCR. **(F-H)** Results from BA inoculation studies in SPF male mice with DSS-induced colitis in the following groups: Vehicle (n=4), BA (n=5), DSS+Vehicle (n=5), DSS+BA (n=4). **(F)** Graph depicting weight loss/gain over time. **(G)** Dot plot depicting colon length. **(H)** Dot plot depicting fold change of *B. acidifaciens* compared to Vehicle control as assessed by PCR. **(I–K)** Results from BA inoculation studies in GF female mice in the following groups: GF control (n=4), GF BA (n=5). **(I)** Graph depicting weight loss/gain over time. **(J)** Dot plot depicting colon length. **(K)** Dot plot depicting fold change of *B. acidifaciens* compared to Vehicle control as assessed by PCR. Error bars equal the standard error mean (SEM). For dot plots, significance was determined using one-way ANOVA and Tukey’s multiple comparisons test. For weight data over time, two-way ANOVA and Dunnett’s multiple comparisons test was used to determine significant (*p<0.05, **p<0.01,***p<0.005, ****p<0.001).

Based on results from the current report, we next wanted to evaluate effects of *B. acidifaciens* on colitis as we previously published it was the most prominent Bacteroides species to be increased during colitis induction, as well as be reduced after treatment with I3C (37). It was noted that while Bacteroides were found to be significantly altered under certain conditions in males and females during DSS colitis, *B. acidifaciens* and other species within this genus were not identified specifically by 16S rRNA sequencing as was previously reported in the TNBS model. This could be attributed to limitations in the sensitivity of the 16S rRNA method identify bacteria at the species and strain levels, as well samples being collected during the “recovery” phase in the DSS model (55). Therefore, studies were conducted to determine if *B. acidifaciens* was significantly altered during the DSS model. As alteration in Bacteroides during colitis and I3C treatment appeared to be more prevalent in females, PCR validation studies investigating *B. acidifaciens* abundance were performed from fecal material collected between AhR*^ΔRorc^* and LM female mice with colitis with or without I3C treatment. Mirroring the 16S rRNA Bacteroides abundance data, female LM mice induced with colitis had a significant increase in *B. acidifaciens* compared to controls, which was reduced with I3C treatment (**Figure 6A**). However, colitis-associated increase of *B. acidifaciens* was not decreased after I3C treatment in AhR*^ΔRorc^* female mice, suggesting the AhR-ILC3-IL22 axis was important in regulating this species during disease. With these results in mind, we investigated the effects of *B. acidifaciens* on DSS-induced colitis in SPF female and male mice as detailed in **Figure 6B** (top). Inoculation of *B. acidifaciens* into disease female mice (DSS+BA) exacerbated colitis severity in females when compared to disease controls (DSS+Vehicle), which was highlighted by significantly increased weight loss (**Figure 6C**) and even more colon shortening (**Figure 6D**). Interestingly, *B. acidifaciens* was also shown to decrease weight and colon length in non-colitis mice (BA) when compared to controls (Vehicle), though it was not found to be statistically significant. As expected based on previous studies, DSS colitis resulted in a significant increase of *B. acidifaciens* in female mice and confirmation of this species successfully engrafting after inoculation was achieved, having higher levels than colitis controls (**Figure 6E**). Unlike female mice, male mice induced with colitis and inoculated with *B. acidifaciens* (DSS+BA) did not have exacerbated disease compared to colitis controls (DSS+Vehicle) as seen with similar, if not in some cases improved, weight loss (**Figure 6F**) and colon shortening (**Figure 6G**). In addition, DSS colitis male mice did not show a significant increase in *B. acidifaciens* when compared to controls, although there was evidence of successful engraftment after inoculation with this species (**Figure 6H**). Lastly, since *B. acidifaciens* exacerbated colitis severity only in females, inoculation of this species into GF female was performed as detailed in **Figure 6B** (bottom). Results showed that *B. acidifaciens* itself was able to induce a colitis-like phenotype in the absence of any other colitis-inducing agent in females, which was illustrated by BA-inoculated GF mice having significantly lower body weight (**Figure 6I**) and colon lengths (**Figure 6J**) compared to GF controls. Successful implantation of *B. acidifaciens* was also confirmed in these studies (**Figure 6K**). Taken altogether, these data suggest *B. acidifaciens* is regulated by AhR expression, particularly in IL-22 producing Rorc-expressing cells such as ILC3s. In addition, the data clearly shows that in the context of colitis, regulation of *B. acidifaciens* appears to be more important in females, as males did not seem to be greatly impacted by increased levels of this species, but females were.

## 4 Discussion

Currently, there is a growing need in the field of IBD-related research to identify and characterize highly effective therapeutics that produce fewer negative side effects as IBD incidence is on the rise worldwide, particularly in western industrialized continents, such as North America and Europe (56). Studies show that the westernization of diets in developing countries has given rise to a higher incidence of IBD, as these diets contain higher levels of protein, sugar, and food additives, which are linked to increased likelihood of developing colitis (57). As one might expect and based on previous studies, consumption of vegetables, such as cruciferous vegetables containing I3C, contain fiber and minerals which can significantly improve IBD symptoms (58, 59). In fact, as with this current report and our past studies (24, 37), there is a growing amount of data showing I3C, and its metabolites, are effective in various models of colitis and intestinal-specific inflammatory disorders (60–64). Many of these reports attribute I3C-mediated protective effects to the ability of this compound to activate AhR, which was illustrated in one of the earliest studies showing I3C’s protective effects against DSS-induced colitis were negated in AhR-null mice (65). Since these early studies, focus has shifted to understanding the cell-specific role of AhR in regulating inflammatory processes that might impact diseases, such as IBDs. In the current report, we continue to identify the AhR-ILC3-IL22 axis as a major pathway to focus on in the context of regulating colitis severity.

Several immune cells express AhR, are present in the GI tract during IBD, and can secrete IL-22, including various innate and adaptive lymphocytes, as well as neutrophils (66). In particular, neutrophils are acknowledged as immune cells that can have both detrimental or positive effects in the context of IBD (67). Previous research by Chen et al. showed that Rorγt, the transcription factor encoded by the Rorc gene, and AhR differentially regulated IL-22 production in neutrophils via the mTOR pathway (68). However, our current report seems to suggest that the majority of IL-22 during colitis, and especially during I3C treatment, does not come from neutrophils, but instead other lymphocyte populations. Another major potential source of IL-22 production is T cell subsets, such as Th22, which are known to produce this cytokine and be regulated by AhR, especially in the context of enteropathogenic bacterial infections (69). However, our current study, adding to our previous report (37), suggests that while IL-22 production increases in response to colitis induction, AhR does not appear to regulate this cytokine to promote any protective effects. Of course, this could be due to several factors, which include the model of colitis, the specific treatment given (e.g. I3C), as well as the inflammatory microenvironment present at the time, particularly whether the condition is acute or chronic in nature. Regardless, our continued research in the area of I3C-mediated protective effects during colitis continues to identify IL-22 production by ILC3s as a major mechanism to be further explored. This is in agreement with other reports which highlight ILC3s as essential cells involved in mediating gut-specific immune responses and the microbial composition within (70). In reference to the latter role of ILC3 in regulating microbial homeostasis, our current report provides evidence the AhR-ILC3-IL22 axis plays a pivotal role in regulating certain microbes that can impact colitis disease.

Based on the 16S rRNA microbial profiles of males and females in the current report, it appears that while there were obvious sex differences, Bacteroides appear to be associated with colitis induction and was regulated by AhR, though more prominently in females. It is important to note that the role of Bacteroides in IBD remains contested as some reports show increases correlate with disease (71, 72), while others report lower levels of Bacteroides are associated with IBD (73). For example, Mills et al. reported an overabundance of *B. vulgatus* was seen in colitis patients and mono-colonization of this species into GF mice induced colitis, which was attributed to proteases produced by this species (74). On the other hand, *B. fragilis* was shown to be protective against colitis and colitis-induced colorectal cancer by TLR2 signaling via polysaccharide A production (75), which was also found to be responsible for ameliorating abnormal metabolism of the anti-fungal agent voriconazole (76). Yan et al. also showed treatment with *B. uniformis* in female mice induced with colitis altered colonic microbiota and bile acid levels to inhibit Th17 differentiation and reduce colitis severity (77). Naturally, the discrepancies in the role of Bacteroides in relation to colitis and general inflammatory disease are likely due in part to different species having different responses. However, other factors could explain these discrepancies, such as sex differences which can impact the host-microbe interactions in response to not only diseases such as colitis, but also response to treatments. Recent reviews focused on the female microbiome also highlight a growing consensus that “healthy” female gut microbiomes tend to have lower abundances of Bacteroides (78). This is best illustrated by our identification of *B. acidifaciens* as having a negative impact on colitis in female mice, but had little effect, if any, in male mice. This seems to be supported partly by recent published results from Zhang et al. which found that *B. acidifaciens* and its extracellular vesicles were protective in the DSS-induced colitis (50). In their report, Zhang et al. found that inoculating *B. acidifaciens* into male mice induced with DSS colitis led to a protective effect, which correlated with a decrease in certain proinflammatory cytokines (e.g. IL-1β, IL-6, and TNF-α) and restoration of tight-junction proteins ZO-1 and occludin. In addition, BA-altered fecal bacteria transplants (FMTs) and fecal filtrate transplants (FTTs) had similar protective outcomes. It is important to note that the report from Zhang et al. only observed this in male mice induced with colitis. In the current report, results did not show a significant improvement in colitis parameters after *B. acidifaciens* inoculation in male mice during DSS-induced colitis. Some factors could help explain the discrepancies in the male mice data from the Zhang et al. report and the current study, which include slight differences in the induction of DSS colitis, but more importantly in the dosing regimen between the studies More specifically, we used 3% DSS to induce colitis in male and female mice, whereas the Zheng et al. report used 2.5% in their male DSS studies. For inoculation studies with *B. acidifaciens*, even though we used the same dose (1 x 10^9^ CFUs), the current report inoculated the species into mice only twice during the experiment (day 0 and day 7). In the Zhang et al. report, researchers gave daily oral administrations of *B. acidifaciens* for 10 days during DSS colitis. These are some of the reasons why we might not have seen as a robust protective response when administrating *B. acidifaciens* in DSS-induced male mice, as was reported by Zhang et al. However, these observations stress the importance of including both sexes in such studies, as there can be drastically different outcomes based solely on the sex of those being evaluated. While the current report does not directly address how *B. acidifaciens* could be contributing to exacerbated colitis severity in females, there is evidence in the previous literature this species has potential pro-inflammatory properties. For example, reports indicate that *B. acidifaciens* can forage and degrade mucus (79, 80). Reduced thickness of the protective mucus layer in the colon can promote gut inflammation and is a known characteristic feature in IBD patients (81). Abnormally high IgA levels are also commonly seen in IBD patients (82), and *B. acidifaciens* has previously been shown to enhance IgA responses in female mice (83). In a report by He et al., results showed *B. acidifaciens* could polarize inflammatory T cells in the gut and exacerbate DSS-induced colitis (84), aligning with results in female mice in the current report. In the initial report from Miyamoto et al. identifying *B. acidifaciens* from the mouse cecum, results showed this species could produce acetic acid (85) which at high levels can induce colitis in animal models (86). These are some potential mechanisms to investigate in future studies directed at better understanding how *B. acidifaciens* could be promoting inflammation during colitis, particularly in females. Current limitations in this report can also be addressed in future studies, such as investigating the effect of *B. acidifaciens* in the TNBS model as well as other models of colitis to determine if this effect is limited to the DSS model. Since *B. acidifaciens* appeared to only exacerbate colitis severity in female mice, we only performed monocolonization studies in GF female mice in the current report. However, in future experiments similar studies in GF male mice should be performed. In addition, given results showing *B. acidifaciens* can induce a colitis-like phenotype in female GF mice, effect of this species on AhR, ILC3s, and IL-22 specifically in this model should be explored.

While the exact role of Bacteroides in colitis, and especially *B. acidifaciens*, will need to be further explored in future studies, past and current reports from our labs repeatedly provide strong evidence that AhR, particularly through the ILC3-IL22 axis, regulates the abundance of Bacteroides during colitis. In our previous publication, we noted that neutralizing IL-22 during TNBS colitis induction in female mice led to significant increases of *B. acidifaciens* which suggested this cytokine was important in regulating abundance of this species in the gut microbiome (37). Species within the Bacteroides genus have also been shown to influence IL-22 production. An example of this would be *B. vulgatus* introduced into recipient mice led to decrease IL-22 secretion, which correlated with disruption in uterine function, insulin resistance, and altered bile acid metabolism (87). The focus of the current report, after identifying ILC3s/T cells as a major source of IL-22 during colitis, was to target AhR deletion in these specific cell types, assess IL-22 production, and determine how this impacted Bacteroides abundance in the gut during colitis. Results showed that this strategy increased Bacteroides in both males and females, further providing evidence of IL-22 involvement in regulating members of this genus. However, future studies blocking IL-22 during colitis in both male and female mice and investigating how this impacts *B. acidifaciens* abundance can provide more definitive proof this cytokine impacts this species specifically. Conducting these studies will be important since a recent report from Mar et al. showed administration of exogenous IL-22 in mice altered the gut microbiome with increased Bacteroides, though this was not in the context of colitis, nor was *B. acidifaciens* levels specifically shown (32). However, it is important to note that other proinflammatory and anti-inflammatory mediators during colitis and activation of AhR can also contribute to the regulation of microbiome, including Bacteroides. We had previously demonstrated that administration of an AhR ligand (I3C) during colitis impacts Th17 and Treg differentiation (37), which are findings supported by others (25, 88, 89). In a more recent report from Jia et al., oral administration of a periodontal pathogen (Porphyromonas gingivalis) can exacerbate colitis, which is characterized by an increase in gut levels of Bacteroides (90). This study highlighted how alterations in the gut microbiome, such as increased Bacteroides, suppressed the linoleic acid pathway, which produces a known activating AhR ligand and resulted in imbalances in the Th17/Treg cell ratio.

Unique to this report were the notable and striking sex differences associated with *B. acidifaciens* impact on colitis. This might not be surprising considering AhR has previously exhibitied sex differences in other studies. For example, exposure to the hallmark AhR ligand, 2,3,7,8-tetrachlorodibenzo-p-dixion (TCDD), has been shown to exert sex-specific alterations in certain genes (91). Some researchers found that certain AhR modulators had sex-specific differences as it related to mitigation of stress and antidepressant effects (40). In another published report, deletion of AhR in the endothelium improved ischemic angiogenesis, though this was only observed specifically in males (92). There are even reports which show that the expression of AhR and AhR-related downstream signaling factors (e.g. Cyp1a1/Cyp1a2) between males and females differ in different organs corresponding to circadian rhythms (93). With these studies in mind, the sex-specific regulation of Bacteroides by AhR seems plausible and could greatly influence how human male and female populations might benefit from AhR-mediated therapeutic targeting, especially those in the IBD patient population. These findings are of importance in terms of future clinical applications with *B. acidifaciens*. Of the few studies specifically investigating *B. acidifaciens* impact on human disease, this species is being promoted as a potential “probiotic”. In addition to the Zhang et al. report showing anti-inflammatory effects of *B. acidifaciens* in a colitis model, this species was shown to have protective effects in other inflammatory and metabolic disorder models. Wang et al. found that reconstituting *B. acidifaciens* into mice was able to make these subjects more resistant to liver injury through a CD95-dependent reduction in hepatocyte apoptosis (94). Yang et al. reported that *B. acidifaciens* had potential as a therapeutic in metabolic diseases such as obesity and diabetes since mice fed with this species gained less weight and fat mass, in addition to having increased serum insulin levels (95). However, all these studies show therapeutic and protective effects in male mice, which ignores any potential sex differences that might arise from using *B. acidifaciens* as a potential probiotic treatment. In the current IBD-related study showing opposite and potentially detrimental effects in female mice where *B. acidifaciens* acts more like a pathobiont, these results present a counterclaim and precaution in the universal characterization of this species as a novel anti-inflammatory probiotic.

## Data Availability

The datasets presented in this study can be found in online repositories. The names of the repository/repositories and accession number(s) can be found below: PRJNA1117471 and PRJNA1117919 (SRA).
