# Supplementary material for: Regulation of Bacteroides acidifaciens by the aryl hydrocarbon receptor in IL-22-producing immune cells has sex-dependent consequential impact on colitis
Source: Front Immunol. 2024 Aug 20;15:1444045. doi: 10.3389/fimmu.2024.1444045 (PMC11368719; doi:10.3389/fimmu.2024.1444045)
Supplement: Supplementary file 1 [file DataSheet1.docx]

Supplementary Material

## Supplementary FiguresSupplementary Figure 1. *Expression of IL-22 in cellular subsets identified in scRNAseq analysis*. Related to Figure 1. Log2 expression of IL-22 was determined using Loupe Browser in the identified cell populations (refer to Figure 1F) from TNBS+Vehicle (top) and TNBS+I3C (bottom) samples.

**Supplementary Figure 2.** *Expression of IL-22ra1 in cellular subsets identified in scRNAseq analysis*. Related to Figure 1. Log2 expression of IL-22ra1 was determined using Loupe Browser in the identified cell populations (refer to Figure 1F) from TNBS+Vehicle (top) and TNBS+I3C (bottom) samples.

**Supplementary Figure 3.** *Expression of IL-10rb in cellular subsets identified in scRNAseq analysis.* Related to Figure 1. Log2 expression of IL-10rb was determined using Loupe Browser in the identified cell populations (refer to Figure 1F) from TNBS+Vehicle (top) and TNBS+I3C (bottom) samples.


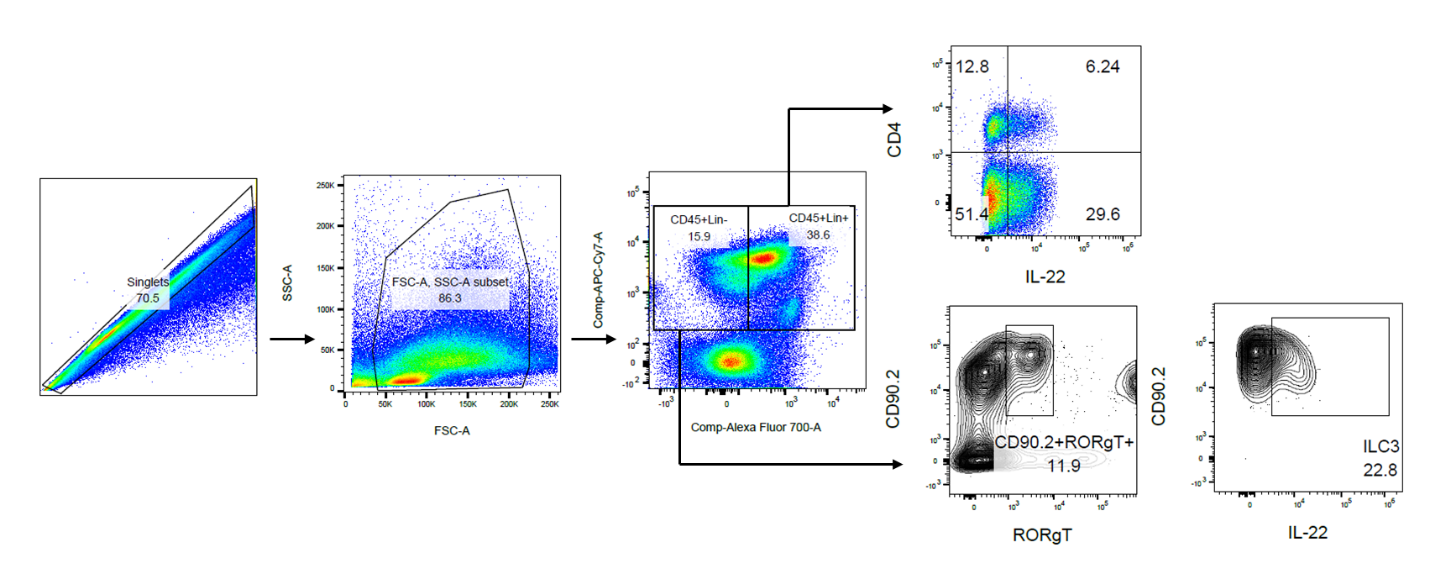


**Supplementary Figure 4.** *Gating strategy for identifying Th22 and IL22-producing ILC3s*. Related to Figure 3. For flow cytometry analysis of Th22 and IL-22-producing immune cells isolated from the lamina propria of experimental mice, events were first gated on singlets (most left) to remove doublets. Next, cells were isolated based on forward scatter (FSC) and side scatter (SSC) to remove potential confounding events (e.g. dead cells, debris). Samples were then sub-divided based on their expression of CD45 and lineage (Lin) markers. For Th22, these cells were defined as being CD45+Lin+CD4+IL-22+. For ILC3s, these cells were identified as being CD45+Lin-, and further gated as being CD90.2+RORgT+. IL-22 producing ILC3s were gated as being IL-22+.
